# Water Pollution Prediction in the Three Gorges Reservoir Area and Countermeasures for Sustainable Development of the Water Environment

**DOI:** 10.3390/ijerph14111307

**Published:** 2017-10-27

**Authors:** Yinghui Li, Shuaijin Huang, Xuexin Qu

**Affiliations:** 1 School of Economic Management, Southwest Jiao tong University, Chengdu 610031, China; 2 Research Center of the Economy of the Upper Reaches of Yangtze River, Chongqing Technology and Business University, Chongqing 400067, China; 3 School of Economics, Chongqing Technology and Business University, Chongqing 400067, China; 17772445691@163.com; 4 Chongqing Key Laboratory of Electronic Commerce & Supply Chain System, Chongqing Technology and Business University, Chongqing 400067, China; q5961339@163.com

**Keywords:** sustainable development, wastewater, pollution, grey prediction, GM (1,1) model, countermeasures

## Abstract

The Three Gorges Project was implemented in 1994 to promote sustainable water resource use and development of the water environment in the Three Gorges Reservoir Area (hereafter “Reservoir Area”). However, massive discharge of wastewater along the river threatens these goals; therefore, this study employs a grey prediction model (GM) to predict the annual emissions of primary pollution sources, including industrial wastewater, domestic wastewater, and oily and domestic wastewater from ships, that influence the Three Gorges Reservoir Area water environment. First, we optimize the initial values of a traditional GM (1,1) model, and build a new GM (1,1) model that minimizes the sum of squares of the relative simulation errors. Second, we use the new GM (1,1) model to simulate historical annual emissions data for the four pollution sources and thereby test the effectiveness of the model. Third, we predict the annual emissions of the four pollution sources in the Three Gorges Reservoir Area for a future period. The prediction results reveal the annual emission trends for the major wastewater types, and indicate the primary sources of water pollution in the Three Gorges Reservoir Area. Based on our predictions, we suggest several countermeasures against water pollution and towards the sustainable development of the water environment in the Three Gorges Reservoir Area.

## 1. Introduction

As the longest river in Asia, the Yangtze River flows through the western, central, and eastern regions of China. It has the largest water freight volume of all global inland rivers, and is known as China’s Golden Waterway, playing an important role in the national economy. The upper reaches of the Yangtze River have abundant mountains, hills, and water resources, providing an ecological shelter for the middle and lower reaches of the river. The middle and lower reaches of the Yangtze River have flat terrain and economic prosperity, but face frequent flood threats. In 1994, the Chinese government initiated the construction of the Three Gorges Project, which was intended to achieve sustainable water environment development, eliminate the flood threats to the middle and lower reaches of the Yangtze River, and promote effective water resource use. The project was designed to fulfill diverse functions including flood prevention, power generation, and shipping [1]. Upon completion in 2009, the project controlled water resources in the upper reaches of the Yangtze River, which covers an area of nearly 1 million km^2^. The government attached substantial importance to the potential environmental impact of this project (including effects on the water environment). In addition to efforts by the State Environmental Protection Administration, teams for coordination and leadership as well as technology implementation were dedicated for the ecological and environmental protection of the Three Gorges Project. The two teams were responsible for ecological and environmental monitoring, the formulation and implementation of special planning, the prevention and control of water pollution in the Three Gorges Reservoir Area (hereafter “Reservoir Area”) and the upper reaches of the Yangtze River, and related follow-up work. So far, the government has spent tens of billions of yuan on the prevention and control of water pollution. Since 2006, stricter policies on energy conservation and discharge reduction have been imposed in China. Efforts for the prevention and control of water pollution in the Three Gorges Reservoir Area were strengthened and total pollutant annual emissions were initially brought under control [2].

The sustainable development of the water environment is necessary to ensure water resource quality, overcome water resource shortages, and maintain a healthy ecological system. These can be achieved through the effective coordination of water pollution prevention and control, as well as water resource use and development, while satisfying the needs of society [3]. The Three Gorges Project promotes the optimal use of water resources. However, the full-load operation of the Three Gorges Dam threatens the sustainable development of the Three Gorges Reservoir Area. Massive wastewater discharge, the primary source of water pollution in the Three Gorges Reservoir Area, along the Yangtze River adversely affects the water resources and the sustainable development of both the Three Gorges Reservoir Area and the entire Yangtze River Basin [4,5]. Therefore, accurate predictions of annual wastewater emission patterns in the Three Gorges Reservoir Area are necessary, to facilitate planning, management, and decision-making related to water pollution in the Three Gorges Reservoir Area.

The current methods commonly used for water pollution prediction can be classified into statistical prediction [6,7,8,9,10], intelligent prediction [11], and mechanism model prediction [12,13,14]. Statistical prediction methods include regression analysis [15,16,17], exponential smoothing prediction [18], and grey system prediction [19,20]. Grey system theory is based on a mixture of known data and unknown information, treating uncertainty and poor information as the research object. System prediction occurs by the formation and development of some known information, the extraction of valuable information, and the correct description of system behavior and evolution. From this description, a quantitative prediction of future changes in intelligence is realized; intelligent prediction methods, using artificial neural network (ANN) prediction, rely on the principles of neural networks [21] and support vector machines (SVM) [22]. SVM is a machine learning method based on Vapnik–Chervonenkis (VC) theory and the structural risk minimization theory of statistical principles, according to the ability to learn and the lack of specific sample pre-error identification. Finally, mechanism model prediction methods are based on the understanding of the characteristics of a real object, analyzing its internal rules, and establishing a model in order to analyze and predict outcomes. Of these analysis types, grey system prediction is highly applicable under the following circumstances: (1) the internal relationships among multiple factors are unclear and (2) data is insufficient or unavailable [23]. Without complete data the behaviors of the system can still be predicted; the calculation is simple and more accurate with less data. At present, the grey model GM (1,1) is widely used for the prediction of hydropower, energy, water pollution levels, etc. [24,25,26]. Wastewater discharge in the Three Gorges Reservoir Area is influenced by a variety of factors, including production mode and the residential styles of the area. However, the causal relationships between these factors and wastewater discharge are complex. The controlling factors are themselves influenced by several factors, and the amount of available data is very limited. Thus, this study employs the GM (1,1) model to predict the annual emissions of the major types of wastewater in the Three Gorges Reservoir Area which severely threaten the sustainable development of the water environment. At present, the researches on water pollution in the three gorges reservoir area are mainly about water quality evaluation and qualitative analysis, but researches on the prediction of water pollution trends using statistical methods are seldom [27,28].

The innovation of this study includes two aspects: method innovation and application innovation: (1) Regarding the method innovation, based on the traditional GM (1,1) model, aiming to minimize the simulation errors, this article takes the method of minimizing error sum of squares between the primitive sequence and the simulation sequence, to established the unconstrained linear programming model of the initial values. Then, the relationship between the initial value and the original sequence and its related parameters is derived by the derivation method and calculation formula of the optimized initial values is also given accordingly. The accuracy of the simulation is improved significantly after the initial values optimization; (2) As for the application innovation, considering that annual wastewater emission in the Three Gorges Reservoir Area is influenced by a variety of factors such as industrial production, life style of the residents; the relationship between these factors and annual wastewater emission is complex; and these influencing factors are themselves influenced by several factors; and the amount of available data is very limited, thus, this study employs the grey even GM (1,1) model to predict the annual emissions of the major types of wastewater in the Three Gorges Reservoir Area from 2015 to 2020. Based on the current and predicted wastewater trends in the study area, we suggest countermeasures against water pollution and towards the sustainable development of the water environment in the Yangtze River.

## 2. Building the Prediction Model

### 2.1. Overview of the Three Gorges Reservoir Area

**Figure 1:**
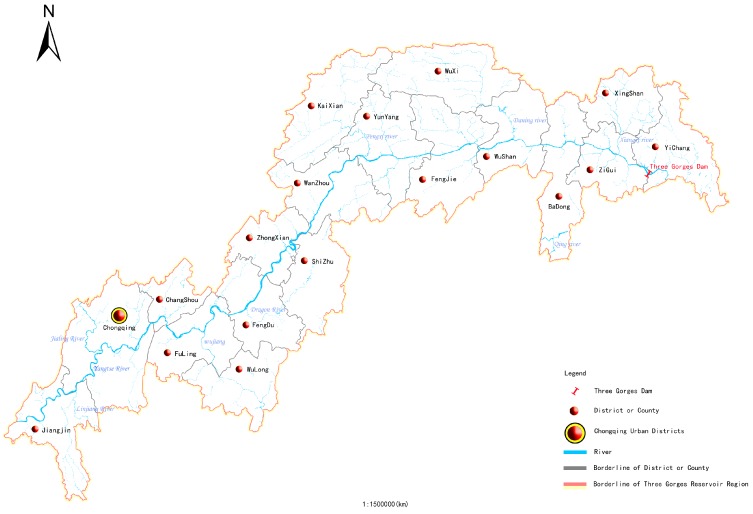
Administrative Map of the Three Gorges Reservoir Area.

The Three Gorges Reservoir Area refers to the reservoir-inundated area influenced by the backwaters, and the administrative region involved in the resettlement of inhabitants when the level of impounded water reaches 175 m. It occupies the lower section of the upper reaches of the Yangtze River (105°44′ to 111°39′ E and 28°30′ to 31°44′ N). Its easternmost part is Yichang, Hubei Province, and its westernmost part is Baxian County, Chongqing Municipality. The Three Gorges Reservoir Area involves a total of 26 districts and/or counties of Chongqing Municipality and Hubei Province. It covers an area of 54,061.5 km^2^, including a water area of 1864 km^2^, accounting for 3.44% of the entire reservoir area, In 2014, the registered population reached 16.9 million, with a total regional GDP of 6320.59 billion yuan and 7487 registered ships. (Shown in Figure 1) [2].

### 2.2. Data Selection and Processing

Based on the principles of data availability and comprehensiveness, we selected original data on four types of wastewater, including industrial wastewater, domestic wastewater, oily wastewater from ships, and domestic wastewater from ships, from the later construction stage of the Three Gorges Reservoir Area, after the reservoir power generation period of 2008 to 2014, to predict annual wastewater emissions from 2015 to 2020. Because industrial wastewater data is limited by subjective and objective factors, such as data acquisition technology and collection conditions, the statistical data differs from the actual situation. Therefore, the model uses *W*_1_ data stabilized by the grey strengthening buffer operator technology. Original data is used for the other three indices (Table 1).

### 2.3. GM (1,1) Modeling

*W*_1_, *W*_2_, *W*_3_ and *W*_4_ indicate the annual emissions of industrial wastewater, domestic wastewater, oily wastewater from ships, and domestic wastewater from ships, respectively, in the Three Gorges Reservoir Area. Based on the above variables and data, building GM (1,1) model to make simulation and prediction, the following steps are needed.

- Step 1: Determine the original sequence:
  (1) $$W_{i}^{\left(0\right)}=\left(w_{i}^{\left(0\right)}\left(1\right),w_{i}^{\left(0\right)}\left(2\right),\cdots ,w_{i}^{\left(0\right)}\left(n\right)\right),i=1,2,3,4$$
  where the subscript “i” denotes the four different kinds of wastewater discharge above, the superscript “(0)” denotes the original sequence and the “n” denotes the sample size of the sequence, the same below. Besides, $W_{i}^{\left(0\right)}$ need to be a non-negative sequence required by GM (1,1) model and our samples all satisfy this requirement, namely:
  (2) $$w_{i}^{\left(0\right)}\left(k\right)\geq 0,\;k=1,2,\cdots ,n$$
- Step 2: Define $W_{i}^{\left(1\right)}$ as the first-order accumulative sequence of $W_{i}^{\left(0\right)}$:
  (3) $$W_{i}^{\left(1\right)}=\left(w_{i}^{\left(1\right)}\left(1\right),w_{i}^{\left(1\right)}\left(2\right),\cdots ,w_{i}^{\left(1\right)}\left(n\right)\right)$$
  where the superscript “(1)” denotes first order accumulation and the samples of the first-order accumulative sequence can be obtained as follows:
  (4) $$w_{i}^{\left(1\right)}\left(k\right)=\sum_{j=1}^{k}w_{i}^{\left(0\right)}\left(j\right),\;k=1,2,\cdots ,n$$
- Step 3: Define $Z_{i}^{\left(1\right)}$ as the mean generation sequence with consecutive neighbors for the first-order accumulative sequence:
  (5) $$Z_{i}^{\left(1\right)}=\left(z_{i}^{\left(1\right)}\left(2\right),z_{i}^{\left(1\right)}\left(3\right),\cdots ,z_{i}^{\left(1\right)}\left(n\right)\right)$$
  where the superscript “(1)” denotes the sequence $Z_{i}^{\left(1\right)}$ is obtained from the first-order accumulative sequence $W_{i}^{\left(1\right)}$. The sample of $Z_{i}^{\left(1\right)}$ can be obtained as follows:
  (6) $$z_{i}^{\left(1\right)}\left(k\right)=\frac{1}{2}\left(w_{i}^{\left(1\right)}\left(k\right)+w_{i}^{\left(1\right)}\left(k-1\right)\right),\;k=2,3,\cdots ,n$$
- Step 4: Then the grey GM (1,1) model for trend prediction of water pollution is formulated as follows:
  (7) $$w_{i}^{\left(0\right)}\left(k\right)+a_{i}z_{i}^{\left(1\right)}\left(k\right)=b_{i},\;i=1,2,3,4,\;k=1,2,\cdots ,n$$
- Step 5: According to Equation (7), parameters *a_i_* and *b_i_* can be calculated by equation:
  (8) $${\left(a_{i},b_{i}\right)}^{T}={\left(B_{i}{}^{T}B_{i}\right)}^{-1}B_{i}{}^{T}Y_{i}$$
  where:
  (9) $$Y_{i}=\left[w_{i}^{\left(0\right)}\left(2\right),w_{i}^{\left(0\right)}\left(3\right),\cdots ,w_{i}^{\left(0\right)}\left(n\right)\right]$$
  (10) $$B_{i}=\left[\begin{array}{cc} -z_{i}^{\left(1\right)}\left(2\right) & 1\\ -z_{i}^{\left(1\right)}\left(3\right) & 1\\ \vdots & \vdots \\ -z_{i}^{\left(1\right)}\left(n\right) & 1 \end{array}\right]$$

Then:

(11) $$B_{i}{}^{T}B_{i}={\left[\begin{array}{cc} -z_{i}^{\left(1\right)}\left(2\right) & 1\\ -z_{i}^{\left(1\right)}\left(3\right) & 1\\ \vdots & \vdots \\ -z_{i}^{\left(1\right)}\left(n\right) & 1 \end{array}\right]}^{T}\left[\begin{array}{cc} -z_{i}^{\left(1\right)}\left(2\right) & 1\\ -z_{i}^{\left(1\right)}\left(3\right) & 1\\ \vdots & \vdots \\ -z_{i}^{\left(1\right)}\left(n\right) & 1 \end{array}\right]=\left[\begin{array}{cc} \sum_{k=2}^{n}{\left[z_{i}^{\left(1\right)}\left(k\right)\right]}^{2} & -\sum_{k=2}^{n}z_{i}^{\left(1\right)}\left(k\right)\\ -\sum_{k=2}^{n}z_{i}^{\left(1\right)}\left(k\right) & n-1 \end{array}\right]$$

(12) $${\left(B_{i}{}^{T}B\right)}^{-1}=\frac{1}{\left(n-1\right)\sum_{k=2}^{n}{\left[z_{i}^{\left(1\right)}\left(k\right)\right]}^{2}-{\left[\sum_{k=2}^{n}z_{i}^{\left(1\right)}\left(k\right)\right]}^{2}}•\left[\begin{array}{cc} n-1 & -\sum_{k=2}^{n}z_{i}^{\left(1\right)}\left(k\right)\\ -\sum_{k=2}^{n}z_{i}^{\left(1\right)}\left(k\right) & \sum_{k=2}^{n}{\left[z_{i}^{\left(1\right)}\left(k\right)\right]}^{2} \end{array}\right]$$

(13) $$B_{i}{}^{T}Y_{i}={\left[\begin{array}{cc} -z_{i}^{\left(1\right)}\left(2\right) & 1\\ -z_{i}^{\left(1\right)}\left(3\right) & 1\\ \vdots & \vdots \\ -z_{i}^{\left(1\right)}\left(n\right) & 1 \end{array}\right]}^{T}\left[\begin{array}{c} w_{i}^{\left(0\right)}\left(2\right)\\ w_{i}^{\left(0\right)}\left(3\right)\\ \vdots \\ w_{i}^{\left(0\right)}\left(n\right) \end{array}\right]=\left[\begin{array}{c} -\sum_{k=2}^{n}w_{i}^{\left(0\right)}\left(k\right)z_{i}^{\left(1\right)}\left(k\right)\\ \sum_{k=2}^{n}w_{i}^{\left(0\right)}\left(k\right) \end{array}\right]$$

Finally, ${\left(a_{i},b_{i}\right)}^{T}$ are calculated as follows:

(14) $$\left[\begin{array}{c} a_{i}\\ b_{i} \end{array}\right]={\left(B_{i}{}^{T}B_{i}\right)}^{-1}B_{i}{}^{T}Y_{i}=\frac{1}{\left(n-1\right)\sum_{k=2}^{n}{\left[z_{i}^{\left(1\right)}\left(k\right)\right]}^{2}-{\left[\sum_{k=2}^{n}z_{i}^{\left(1\right)}\left(k\right)\right]}^{2}}\;\times \left[\begin{array}{c} -\left(n-1\right)\sum_{k=2}^{n}x_{i}^{\left(0\right)}\left(k\right)z_{i}^{\left(1\right)}\left(k\right)+\sum_{k=2}^{n}x_{i}^{\left(0\right)}\left(k\right)\sum_{k=2}^{n}z_{i}^{\left(1\right)}\left(k\right)\\ -\sum_{k=2}^{n}z_{i}^{\left(1\right)}\left(k\right)\sum_{k=2}^{n}x_{i}^{\left(0\right)}\left(k\right)z_{i}^{\left(1\right)}\left(k\right)+\sum_{k=2}^{n}x_{i}^{\left(0\right)}\left(k\right)\sum_{k=2}^{n}{\left[z_{i}^{\left(1\right)}\left(k\right)\right]}^{2} \end{array}\right]\;=\left[\begin{array}{c} \frac{\frac{1}{n-1}\sum_{k=2}^{n}x_{i}^{\left(0\right)}\left(k\right)\sum_{k=2}^{n}z_{i}^{\left(1\right)}\left(k\right)-\sum_{k=2}^{n}x_{i}^{\left(0\right)}\left(k\right)z_{i}^{\left(1\right)}\left(k\right)}{\sum_{k=2}^{n}{\left[z_{i}^{\left(1\right)}\left(k\right)\right]}^{2}-\frac{1}{n-1}{\left[\sum_{k=2}^{n}z_{i}^{\left(1\right)}\left(k\right)\right]}^{2}}\\ \frac{1}{n-1}[\sum_{k=2}^{n}x_{i}^{\left(0\right)}\left(k\right)+a\sum_{k=2}^{n}z_{i}^{\left(1\right)}\left(k\right)] \end{array}\right]$$

- Step 6: Define the winterization (or image) equation of the grey differential Equation (7) as follows:
  (15) $$\frac{dw_{i}^{\left(1\right)}(t)}{dt}+a_{i}w_{i}^{\left(1\right)}(t)=b_{i}$$
- Step 7: With the values of *a_i_* and *b_i_* , the solution of whitenization Function (15) is given by:
  (16) $${\hat{w}}_{i}^{\left(1\right)}\left(t\right)=\left(w_{i}^{\left(0\right)}\left(1\right)-\frac{b_{i}}{a_{i}}\right)e^{-a_{i}(t-1)}+\frac{b_{i}}{a_{i}}$$
- Step 8: Correspondingly, when t = k, the discrete form of Function (16) is given by:
  (17) $${\hat{w}}_{i}^{\left(1\right)}\left(k\right)=\left(w_{i}^{\left(0\right)}\left(1\right)-\frac{b_{i}}{a_{i}}\right)e^{-a_{i}(k-1)}+\frac{b_{i}}{a_{i}},\;k=1,2,\cdots ,n$$

Function (17) is called time response function of GM (1,1) model (7).

- Step 9: Finally, the reduction value can be calculated:
  (18) $$\begin{array}{l} {\hat{w}}_{i}^{\left(0\right)}\left(k\right)=\alpha_{i}^{\left(1\right)}{\hat{w}}_{i}^{\left(1\right)}\left(k\right)={\hat{w}}_{i}^{\left(1\right)}\left(k\right)-{\hat{w}}_{i}^{\left(1\right)}\left(k-1\right)\\ =\left(1-e^{a_{i}}\right)\left(w_{i}^{\left(0\right)}\left(1\right)-\frac{b_{i}}{a_{i}}\right)e^{-a_{i}(k-1)},k=1,2,\cdots ,n \end{array}$$

With Equation (18), we can make grey simulation and prediction of the annual emissions of waste water in the Three Gorges Reservoir Area.

However, during model calculation, the statistical data of certain indexes differs somewhat from the actual index values because of various subjective or objective factors. Here, we process these differences by using the grey strengthening buffer operator technology based on the following principle. Assume that the original sequence and its buffer sequence are as follows:

(19) X=(x(1),x(2),⋯,x(n))

(20) XD=(x(1)d,x(2)d,⋯,x(n)d)

where:

(21) $$x\left(k\right)d=\frac{x\left(1\right)+x\left(2\right)+\cdots +x\left(k-1\right)+kx\left(k\right)}{2k-1},k=1,2,\cdots ,n-1$$

(22) x(n)d=x(n)

when *X* is a monotonic increasing sequence or monotonic attenuating sequence, *D* is a strengthening operator.

### 2.4. Principle for Optimizing Initial Values of the GM (1,1) Model

In the process of grey prediction modeling, $w_{i}^{\left(0\right)}\left(1\right)$ is used as an initial value to derive the time response function of the original sequence; namely, a formula with $w_{i}^{\left(0\right)}\left(1\right)$ is used to denote ${\hat{w}}_{i}^{\left(1\right)}\left(k\right)$. The fitting curve obtained in this way is assumed to pass through the point $\left(1,w_{i}^{\left(0\right)}\left(1\right)\right)$ in the coordinate plane. Based on the principle of least squares, however, it is unreasonable to use $w_{i}^{\left(0\right)}\left(1\right)$ as an initial value to derive ${\hat{w}}_{i}^{\left(1\right)}\left(k\right)$; i.e., the fitting curve does not necessarily pass through the point $\left(1,w_{i}^{\left(0\right)}\left(1\right)\right)$, and ${\hat{w}}_{i}^{\left(1\right)}\left(k\right)$ is not correlated with the initial values of the original sequence. In other words, the grey prediction model discards the role of $w_{i}^{\left(0\right)}\left(1\right)$.

In order to address the issue above, this study optimizes the initial values of the time response sequence (17) based on minimizing the sum of squares of the errors between the original sequences. The detailed optimization principle and procedure are as follows: According to Equation (17), obtain ${\hat{w}}_{i}^{\left(1\right)}\left(1\right)=w_{i}^{\left(1\right)}\left(1\right)=w_{i}^{\left(0\right)}\left(1\right)$. Make ${\hat{w}}_{i}^{\left(1\right)}\left(1\right)=w_{i}^{\left(1\right)}\left(1\right)=w_{i}^{\left(0\right)}\left(1\right)=C_{i}$. Then, the reduction value sequence can be expressed as follows:

(23) $${\hat{w}}_{i}^{\left(0\right)}\left(k\right)=\left(1-e^{a_{i}}\right)\left(C_{i}-\frac{b_{i}}{a_{i}}\right)e^{-a_{i}\left(k-1\right)},\;k=1,2,\cdots ,n$$

According to Equation (23), the following result is obtained:

(24) $${\hat{w}}_{i}^{\left(0\right)}(1)=(1-e^{a_{i}})(C_{i}-\frac{b_{i}}{a_{i}})$$

(25) $${\hat{w}}_{i}^{\left(0\right)}\left(2\right)=\left(1-e^{a_{i}}\right)\left(C_{i}-\frac{b_{i}}{a_{i}}\right)e^{-a_{i}}$$

⋮

(26) $${\hat{w}}_{i}^{\left(0\right)}\left(n\right)=\left(1-e^{a_{i}}\right)\left(C_{i}-\frac{b_{i}}{a_{i}}\right)e^{-a_{i}\left(n-1\right)}$$

Define ${\tilde{\Delta }}_{i}\left(k\right)$, k=1,2,⋯,n as the square sequence about the relative simulation errors;

Then:

(27) $${\tilde{\Delta }}_{i}\left(1\right)={\left[\frac{{\hat{w}}_{i}^{\left(0\right)}\left(1\right)-w_{i}^{\left(0\right)}\left(1\right)}{w_{i}^{\left(0\right)}\left(1\right)}\right]}^{2}={\left[\frac{\left(1-e^{a_{i}}\right)\left(C_{i}-\frac{b_{i}}{a_{i}}\right)-w_{i}^{\left(0\right)}\left(1\right)}{w_{i}^{\left(0\right)}\left(1\right)}\right]}^{2}$$

(28) $${\tilde{\Delta }}_{i}\left(2\right)={\left[\frac{{\hat{w}}_{i}^{\left(0\right)}\left(2\right)-w_{i}^{\left(0\right)}\left(2\right)}{w_{i}^{\left(0\right)}\left(2\right)}\right]}^{2}={\left[\frac{\left(1-e^{a_{i}}\right)\left(C_{i}-\frac{b_{i}}{a_{i}}\right)e^{-a_{i}}-w_{i}^{\left(0\right)}\left(2\right)}{w_{i}^{\left(0\right)}\left(2\right)}\right]}^{2}$$

⋮

(29) $${\tilde{\Delta }}_{i}\left(n\right)={\left[\frac{{\hat{w}}_{i}^{\left(0\right)}\left(n\right)-w_{i}^{\left(0\right)}\left(n\right)}{w_{i}^{\left(0\right)}\left(n\right)}\right]}^{2}={\left[\frac{\left(1-e^{a_{i}}\right)\left(C_{i}-\frac{b_{i}}{a_{i}}\right)e^{-a_{i}(n-1)}-w_{i}^{\left(0\right)}\left(n\right)}{w_{i}^{\left(0\right)}\left(n\right)}\right]}^{2}$$

Define ${\tilde{\Delta }}_{i}$ as the sum of squares about the relative simulation errors;

Then:

(30) $${\tilde{\Delta }}_{i}={\tilde{\Delta }}_{i1}+{\tilde{\Delta }}_{i2}+\cdots +{\tilde{\Delta }}_{in}$$

Based on the minimized sum of relative simulation errors, and to obtain good simulation accuracy, $C_{i}$ must comply with the following unconstrained linear programming model:

(31) $$\min{\tilde{\Delta }}_{i}={\tilde{\Delta }}_{i1}+{\tilde{\Delta }}_{i2}+\cdots +{\tilde{\Delta }}_{in}$$

When ${\tilde{\Delta }}_{i}$ is minimized, $C_{i}$ obtains the optimal value, namely:

(32) $$\frac{d{\tilde{\Delta }}_{i}}{dC_{i}}=\frac{d{\tilde{\Delta }}_{i1}}{dC_{i}}+\frac{d{\tilde{\Delta }}_{i2}}{dC_{i}}+\cdots +\frac{d{\tilde{\Delta }}_{in}}{dC_{i}}=0$$

(33) $$\frac{d{\tilde{\Delta }}_{i1}}{dC_{i}}=2\left[{\left(\frac{1-e^{2a_{i}-a_{i}}}{w_{i}^{\left(0\right)}\left(1\right)}\right)}^{2}●C_{i}-\frac{{\left(1-e^{2a_{i}-a_{i}}\right)}^{2}\frac{b_{i}}{a_{i}}+\left(1-e^{2a_{i}-a_{i}}\right)w_{i}^{\left(0\right)}\left(1\right)}{{\left[w_{i}^{\left(0\right)}\left(1\right)\right]}^{2}}\right]$$

(34) $$\frac{d{\tilde{\Delta }}_{i2}}{dC_{i}}=2\left[{\left(\frac{e^{-a_{i}}-1}{w_{i}^{\left(0\right)}\left(2\right)}\right)}^{2}●C_{i}-\frac{{\left(e^{-a_{i}}-1\right)}^{2}\frac{b_{i}}{a_{i}}+\left(e^{-a_{i}}-1\right)w_{i}^{\left(0\right)}\left(2\right)}{{\left[w_{i}^{\left(0\right)}\left(2\right)\right]}^{2}}\right]$$

⋮

(35) $$\frac{d{\tilde{\Delta }}_{in}}{dC_{i}}=2\left[{\left(\frac{e^{-a_{i}(n-1)}-e^{2a_{i}-a_{i}n}}{w_{i}^{\left(0\right)}\left(n\right)}\right)}^{2}●C_{i}-\frac{{\left(e^{-a_{i}(n-1)}-e^{2a_{i}-a_{i}n}\right)}^{2}\frac{b_{i}}{a_{i}}+\left(e^{-a_{i}(n-1)}-e^{2a_{i}-a_{i}n}\right)w_{i}^{\left(0\right)}\left(n\right)}{{\left[w_{i}^{\left(0\right)}\left(n\right)\right]}^{2}}\right]$$

According to Equation (5) and $\frac{d{\tilde{\Delta }}_{i1}}{dC_{i}}$, $\frac{d{\tilde{\Delta }}_{i2}}{dC_{i}}$, ⋯, $\frac{d{\tilde{\Delta }}_{in}}{dC_{i}}$ the optimized original value $C_{i}^{*}$ can be obtained:

(36) $$C_{i}^{*}=\frac{\sum_{j=2}^{n}\frac{{\left(e^{-a_{i}(n-1)}-e^{2a_{i}-a_{i}n}\right)}^{2}\frac{b_{i}}{a_{i}}+\left(e^{-a_{i}(n-1)}-e^{2a_{i}-a_{i}n}\right)w_{i}^{\left(0\right)}\left(n\right)}{{\left[w_{i}^{\left(0\right)}\left(n\right)\right]}^{2}}}{{\sum_{j=2}^{n}\left(\frac{e^{-a_{i}(n-1)}-e^{2a_{i}-a_{i}n}}{w_{i}^{\left(0\right)}\left(n\right)}\right)}^{2}}$$

### 2.5. Test Method for the GM (1,1) Model

The effectiveness of the above model must be tested [29,30]. For the grey GM (1,1) model, we verify its effectiveness through an average relative error test and a grey correlation degree test. The test principle and procedure are as follows. Regarding prediction of the water environment of the Three Gorges Reservoir Area, the original sequence is:

(37) $$W_{i}^{\left(0\right)}=\left(w_{i}^{\left(0\right)}(1),w_{i}^{\left(0\right)}(2),\cdots ,w_{i}^{\left(0\right)}(n)\right)$$

The corresponding analog sequence is:

(38) $${\hat{W}}_{i}^{\left(0\right)}=\left({\hat{w}}_{i}^{\left(0\right)}(1),{\hat{w}}_{i}^{\left(0\right)}(2),\cdots ,{\hat{w}}_{i}^{\left(0\right)}(n)\right)$$

We obtain the residual error sequence as follows:

(39) $$\varepsilon_{i}^{\left(0\right)}=\left(\varepsilon_{i}^{\left(0\right)}\left(1\right),\varepsilon_{i}^{\left(0\right)}\left(2\right),\cdots ,\varepsilon_{i}^{\left(0\right)}\left(n\right)\right)=\left(w_{i}^{\left(0\right)}\left(1\right)-{\hat{w}}_{i}^{\left(0\right)}\left(1\right),w_{i}^{\left(0\right)}\left(2\right)-{\hat{w}}_{i}^{\left(0\right)}\left(2\right),\cdots ,w_{i}^{\left(0\right)}\left(n\right)-{\hat{w}}_{i}^{\left(0\right)}\left(n\right)\right)$$

#### 2.5.1. Average Relative Error Test

The relative error sequence is:

(40) $$\Delta_{i}\left(k\right)=\left|\frac{\varepsilon_{i}^{\left(0\right)}\left(k\right)}{w_{i}^{\left(0\right)}\left(k\right)}\right|,k=1,2,\cdots ,n$$

The average relative error can be expressed as:

(41) $${\bar{\Delta }}_{i}=\frac{1}{n}\sum_{k=1}^{n}\Delta_{i}\left(k\right),k=1,2,\cdots ,n$$

Smaller values of average relative error, correspond to better simulation effects of the model and greater accuracy in prediction results. We specify the constant *c*. If ${\bar{\Delta }}_{i}\leq c$, the model is called a residual error qualified model.

#### 2.5.2. Grey Correlation Degree Test

Make:

(42) $$\left|s_{i}\right|=\left|\sum_{k=2}^{n-1}[w_{i}^{\left(0\right)}\left(k\right)-w_{i}^{\left(0\right)}\left(1\right)]+\frac{1}{2}[w_{i}^{\left(0\right)}\left(n\right)-w_{i}^{\left(0\right)}\left(1\right)]\right|,\;k=1,2,\cdots ,n$$

(43) $$\left|{\hat{s}}_{i}\right|=\left|\sum_{k=2}^{n-1}[{\hat{w}}_{i}^{\left(0\right)}\left(k\right)-{\hat{w}}_{i}^{\left(0\right)}\left(1\right)]+\frac{1}{2}[{\hat{w}}_{i}^{\left(0\right)}\left(n\right)-{\hat{w}}_{i}^{\left(0\right)}\left(1\right)]\right|,\;k=1,2,\cdots ,n$$

The grey absolute degree of correlation between the original sequence $W_{i}^{\left(0\right)}$ and the analog sequence ${\hat{W}}_{i}^{\left(0\right)}$ is defined as follows:

(44) $$\eta_{i}=\frac{1+\left|s_{i}\right|+\left|{\hat{s}}_{i}\right|}{1+\left|s_{i}\right|+\left|{\hat{s}}_{i}\right|+\left|{\hat{s}}_{i}-s_{i}\right|},\eta_{i}\left(0\leq \eta_{i}\leq 1\right)$$

As the $\eta_{i}$ value approaches 1, the simulation effect of the model improves and accuracy of the prediction results increases. We specify a constant $\eta_{0}\left(0\leq \eta_{0}\leq 1\right)$. If $\eta_{i}\geq \eta_{0}$, the model is called a qualified model about the grey correlation degree [31]. The commonly used levels of accuracy are given in Table 2:

## 3. Trend Prediction and Analysis of Water Pollution in the Three Gorges Reservoir Area

### 3.1. GM (1,1) Model Simulation and Test Using Historical Data

The original data sequence of $W_{1},W_{2},W_{3},W_{4}$ is applied to the model, and the original data is simulated and tested. We use $W_{1}$ as an example. To use the GM (1,1) model for simulated calculation, we follow the steps below:

- Step 1: Determine the original sequence:
  (45) $$W_{1}^{\left(0\right)}=\left(5.58,\;5.10,\;4.00,\;3.04,\;2.69,\;2.61,\;2.12\right)$$
- Step 2: Calculate the first-order accumulative sequence of $W_{1}^{\left(0\right)}$:
  (46) $$W_{1}^{\left(1\right)}=\;\left(5.58,\;10.68,\;14.68,\;17.72,\;20.41,\;23.02,\;25.14\right)$$
- Step 3: Calculate the mean generation sequence with consecutive neighbors regarding $W_{1}^{\left(0\right)}$:
  (47) $$Z_{1}^{\left(1\right)}=\;\left(8.13,\;12.68,\;16.20,\;19.07,\;21.72,\;24.08\right)$$
- Step 4: Formulate the grey GM (1,1) model for $W_{1}$:
  (48) $$w_{1}^{\left(0\right)}\left(k\right)+a_{1}z_{1}^{\left(1\right)}\left(k\right)=b_{1},\;k=1,2,\cdots ,7$$
- Step 5: Calculate the values of the parameters $a_{1}$ and $b_{1}$ to be estimated:
  (49) $$a_{1}=0.181\;b_{1}=6.338$$
- Step 6: Write the whitening differential equation of Equation (49):
  (50) $$\frac{dw_{1}^{\left(1\right)}(t)}{dt}+0.181w_{1}^{\left(1\right)}(t)=6.338$$
- Step 7: The solution of whitening Function (50) is as follows:
  (51) $${\hat{w}}_{1}^{\left(1\right)}\left(t\right)=\left(w_{1}^{\left(0\right)}\left(1\right)-35.02\right)e^{-0.181(t-1)}+35.02$$
- Step 8: Write the discrete form of Function (51):
  (52) $${\hat{w}}_{1}^{\left(1\right)}\left(k\right)=\left(w_{1}^{\left(0\right)}\left(1\right)-35.02\right)e^{-0.181(k-1)}+35.02,\;k=1,2,\cdots ,7$$
- Step 9: Determine the reduction value sequence as follows:
  (53) $${\hat{w}}_{1}^{\left(0\right)}\left(k\right)=\left(1-e^{0.181}\right)\left(5.58-35.02\right)e^{-0.181(k-1)},k=1,2,\cdots ,7$$

Likewise, determine the reduction value sequences of $W_{2},W_{3},W_{4}$ as follows:

(54) $${\hat{w}}_{2}^{\left(0\right)}\left(k\right)=\left(1-e^{-0.056}\right)\left(5.93+100.66\right)e^{0.056(k-1)},k=1,2,\cdots ,7$$

where:

(55) $$a_{2}=-0.056\;b_{2}=5.637$$

(56) $${\hat{w}}_{3}^{\left(0\right)}\left(k\right)=\left(1-e^{-0.011}\right)\left(41.20+4114\right)e^{0.011(k-1)},k=1,2,\cdots ,7$$

where:

(57) $$a_{3}=-0.011\;b_{3}=45.254$$

(58) $${\hat{w}}_{4}^{\left(0\right)}\left(k\right)=\left(1-e^{0.01}\right)\left(404.60-40851.4\right)e^{-0.01(k-1)},k=1,2,\cdots ,7$$

where:

(59) $$a_{4}=0.010\;b_{4}=408.514$$

According to Equation (6), MATLAB2015 is used to calculate the optimized initial values of the GM (1,1) model as follows:

(60) $$C_{1}^{*}=5.59,\;C_{2}^{*}=5.93,\;C_{3}^{*}=41.16,\;C_{4}^{*}=404.64$$

The optimized reduction value sequences of the GM (1,1) model are calculated as follows:

(61) $${\hat{w}}_{1}^{\left(0\right)}\left(k\right)=\left(1-e^{0.181}\right)\left(5.59-35.02\right)e^{-0.181(k-1)},k=1,2,\cdots ,7$$

(62) $${\hat{w}}_{2}^{\left(0\right)}\left(k\right)=\left(1-e^{-0.056}\right)\left(5.93+100.66\right)e^{0.056(k-1)},k=1,2,\cdots ,7$$

(63) $${\hat{w}}_{3}^{\left(0\right)}\left(k\right)=\left(1-e^{-0.011}\right)\left(41.16+4114\right)e^{0.011(k-1)},k=1,2,\cdots ,7$$

(64) $${\hat{w}}_{4}^{\left(0\right)}\left(k\right)=\left(1-e^{0.01}\right)\left(404.64-40851.4\right)e^{-0.01(k-1)},k=1,2,\cdots ,7$$

According to Equations (61)–(64), the simulation values and their errors are determined as listed in Table 3, Table 4, Table 5 and Table 6.

According to Table 2, using Table 3, Table 4, Table 5 and Table 6 and Equations (40)–(44), the results of the average relative error test and grey correlation degree test for the simulation results of *W*_1_, *W*_2_, *W*_3_ and *W*_4_ are shown in Table 7. According to the average relative error, the simulation accuracies for *W*_1_, *W*_2_, *W*_3_ and *W*_4_ are all above Grade 3. According to the grey correlation degree test, all simulation accuracies are Grade 1. This further shows that the GM (1,1) model is highly effective and suitable to predict water pollution in the Three Gorges Reservoir Area.

### 3.2. Results of GM (1,1) Model Prediction

**Figure 2:**
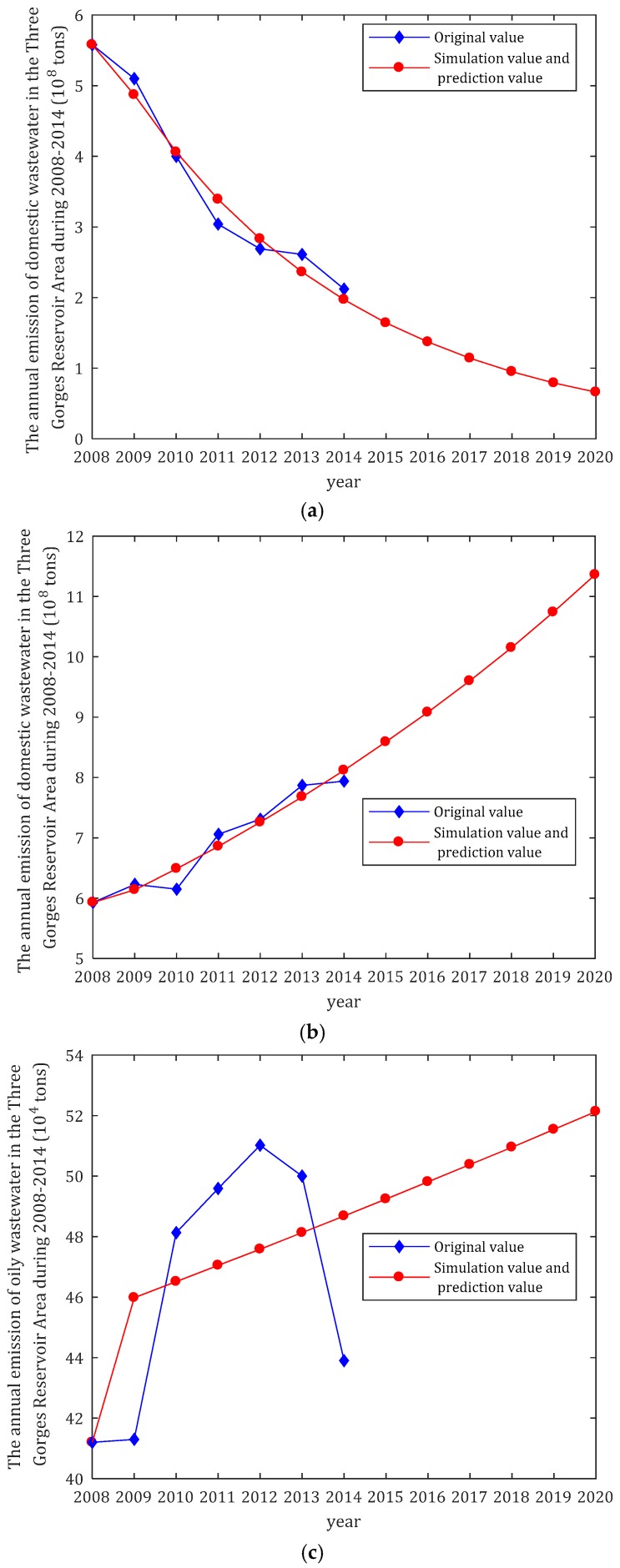

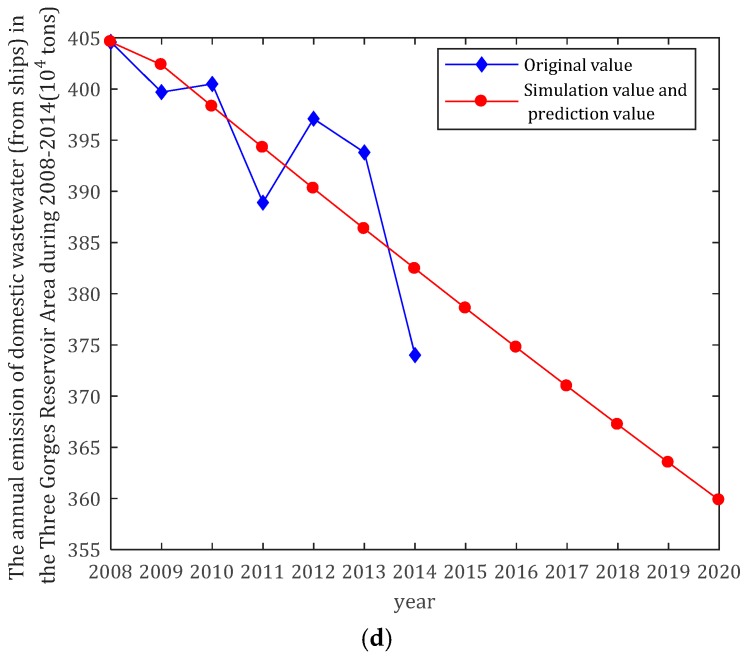
The curves of original, simulated and predicted values of (**a**) W_1_; (**b**) W_2_; (**c**) W_3_ and (**d**) W_4_.

Using Equations (12)–(15), the optimized GM (1,1) model predicted the annual emission values for the four different types of wastewater in the Three Gorges Reservoir Area during the years 2015 to 2020 (Table 8). The following trends are revealed: (1) the annual emission of industrial wastewater decreases each year, with an annual average decrease of 15.1%; (2) the annual emission of domestic wastewater increases each year, with an annual average increase of 5.6%; (3) the annual emission of oily wastewater from ships increases more slowly, with an annual average increase of 2.2%; and (4) the annual emission of domestic wastewater from ships steadily decreases, with an annual average decrease of 1% (Figure 2).

### 3.3. Analysis of Water Pollution Trend Prediction Results

#### 3.3.1. Industrial Wastewater Discharge

The continuous decrease in industrial wastewater annual emission is likely due to efforts at all levels of government in the Three Gorges Reservoir Area; these appear to have controlled industrial wastewater pollution. For example, serious measures are employed to strengthen ecological protection in the river basin and to manage the discharge and treatment of wastewater from businesses along the Yangtze River. However, the construction of industrial parks in the Three Gorges Reservoir Area involves various problems, including relatively small-scale, disperse pollutant distributions, and delays in the construction of the industrial ecological chain. Therefore, pollution control is not highly efficient and industrial wastewater annual emission could increase again in the future.

#### 3.3.2. Domestic Wastewater Discharge

The continuous increase in domestic wastewater annual emission in the Three Gorges Reservoir Area is attributed to two main reasons: (1) the continued increase in the population of the Three Gorges Reservoir Area (the registered population increased from 20,679,000 in 2008 to 21,528,000 in 2014) at a very high population density; and (2) municipal sewage treatment plants have issues including outdated pipe networks, low wastewater collection rates, and poor nitrogen and phosphorus removal capabilities. In addition, the sewage treatment process is unsuitable for the actual conditions of some small towns, and sewage treatment plants and waste landfill sites are severely inadequate in some new urban areas.

#### 3.3.3. Shipping Industry Wastewater Discharge

Pollutants discharged by ships mainly include oily wastewater and domestic wastewater. The decrease in shipping industry wastewater is closely related to recent measures taken by the maritime authority and port and shipping enterprises in China. These include joint management and control, and special departments that dispose of oily wastewater, residual oil, and garbage discharged from ships. The growth trend of ship oil pollution water showed an inflection point in 2012, attributed to the state of the Chinese economy in 2012 before the high-speed development of freight demand continued growth. The Three Gorges Reservoir area registered ship total reached 8215 ships in 2012; since then, affected by the economic downward pressure, the ships registered in 2013 were reduced to 7937 ships and in 2014 to 7487 ships, which is reflected by the decreased oil content decreased after the first year. However, the completion of the Three Gorges Reservoir enhanced the shipping capacity of the Three Gorges Reservoir Area. With the construction of the Yangtze River Economic Zone and signs of economic rebound in China, the shipping industry of the Three Gorges Reservoir Area is expected to become more prosperous. Thus, pollutants discharged during daily ship operation and by various pollution accidents will inevitably affect water quality and threaten the safety of the water environment throughout the Reservoir Area.

## 4. Countermeasures against Water Pollution in the Three Gorges Reservoir Area

According to our predictions of annual wastewater emissions in the Three Gorges Reservoir Area over the next six years, emissions of the four major wastewater sources are decreased year by year. However, economic development and population growth may affect wastewater discharge patterns and thereby the sustainability of the water environment in the Three Gorges Reservoir area. To ensure future sustainable development of the water environment, further control the reduction of wastewater emissions, and regulate industrial wastewater and wastewater from ships, we propose the following countermeasures against water pollution.

### 4.1. Strengthen Governmental Responsibility

According to the theory of public economics, the water environment of the Three Gorges Reservoir Area has the characteristics of a public product, and can be considered a national public product owned by all Chinese people. The residents, enterprises, and local governments in the Three Gorges Reservoir Area are direct producers of this public product. However, this public product has a wide scope of beneficiaries, so its costs should be assumed by all Chinese citizens. Governments possess the total property rights to the water environment of the Three Gorges Reservoir Area and are duty-bound to administer it. Therefore, governments should play an intermediary role in various socioeconomic activities by: (1) coordinating and optimizing the actions of related enterprises, residents, and local governments from a holistic perspective; (2) ensuring that all users of this public product assume its production costs; and (3) providing this public product through selective incentives, so that paying individuals can derive appropriate benefits [32], thus developing an administration mechanism that favors the sustainable development of the water environment of the Three Gorges Reservoir Area.

### 4.2. Reconstruct Residential Spaces for Urban and Rural Residents

In order to effectively control the influence of wastewater on the water environment in the Three Gorges Reservoir area, residential spaces in the Reservoir Area should be optimized to share community resources. Residential spaces in the Three Gorges Reservoir area should follow the natural ecological system, optimize the layout of urban and rural industries, adjust modes of industrial production and living, and realize the organic coupling of production and resident life activities. Moreover, the following measures are vital: (1) optimize the functions of central cities, and improve urban living environments (for example, accelerate the construction of treatment facilities for urban sewage, industrial wastewater, and garbage); (2) optimize industrial practices and cluster industries and the population according to the ecological conditions, location, and industrial characteristics of different towns; and (3) promote the construction of ecologically attractive villages and improve the integration of industrial and social activities in rural areas.

### 4.3. Develop an Ecological Industry Model

In order to reduce the production of industrial wastewater, the industrial development of the Three Gorges Reservoir area should adopt the “eco industrial model” in the concept of the circular economy. The eco-industrial chain enables a balance between the protection of the ecological environment and the development of the economy. In the Three Gorges Reservoir Area, the following measures are necessary: (1) promote clean production and green corporate culture among manufacturing enterprises and transform the high-consumption, high-emission development patterns into resource-conserving, environmentally friendly patterns; (2) build industrial parks comprising business clusters according to eco-industrial park standards and develop a circular industry network for resources, energy, and wastes [33]; (3) redesign existing eco-industrial parks and other economic development zones according to circular economic concepts [34]. These measures will enable the sharing of infrastructure, public resources, and energy, the continuous improvement of clean production and environmental management systems, and the optimized overall ecological efficiency of each industrial park. The goal is the adoption of eco-industrial development practices.

### 4.4. Improve Ship Pollution Prevention and Control Systems in the Three Gorges Reservoir Area

In order to effectively prevent and control wastewater pollution threat caused by the booming shipping industry in the Three Gorges Reservoir area, scientific planning and optimized working practices are necessary. Specifically, this requires: (1) improved supervision of the pollution prevention and control system and identification of the key polluted areas, major types of pollutants, and high pollution periods [35]; (2) an emergency response system, inter-departmental cooperation mechanisms, and continuous improvement of anti-pollution equipment; (3) a ship pollutant reception and disposal system and zero-discharge mode characterized by “storage on board and disposal on shore”; (4) a ship pollutant monitoring system and whole-process supervision of ship pollutants (including generation, storage, and onshore disposal); (5) strengthened publicity and education about water environment protection and enhanced environmental consciousness and responsibility among personnel operating at sea.

## 5. Conclusions

This study presents a grey GM (1,1) model with optimized initial values, which is used to predict the annual emissions of four types of wastewater in the Three Gorges Reservoir Area during the 13th Five-year Period (2016 to 2020). According to the prediction results, the discharge of industrial wastewater is successfully managed, that from ships has decreased slightly, domestic wastewater annual emission has increased each year, and the total annual emission of wastewater has not increased significantly. Overall, wastewater pollution severely threatens the water environment of the Three Gorges Reservoir Area and affects its sustainable socioeconomic development. To prevent further increases in water pollution and ensure sustainable development of the water environment in the Yangtze River basin, we proposed several appropriate measures, for example, strengthening governmental responsibility, reconstructing residential spaces, optimizing industrial layouts, and developing new industrial and social practices.

However, water pollution data in this region is limited for several reasons, including the short history of the Three Gorges Reservoir Area, difficulty in data acquisition, and poor data continuity. In future, the grey GM (1,1) model will be further optimized to expand the prediction coverage related to wastewater discharge indexes. The goal of such studies is to survey the state of water pollution in the Reservoir Area more comprehensively and scientifically and to provide a decision-making basis for all levels of government in China regarding the sustainable development of the water environment.

## Figures and Tables

**Table 1:** Annual emissions of main water pollutants in the Three Gorges Reservoir Area between 2008 and 2014.

Year	$W_{1}$ (10^8^ Tons)		$W_{2}$ (10^8^ Tons)	$W_{3}$ (10^4^ Tons)	$W_{4}$ (10^4^ Tons)
	X	XD			
2008	5.58	5.58	5.93	41.20	404.6
2009	4.86	5.10	6.23	41.30	399.7
2010	3.19	4.00	6.15	48.13	400.5
2011	1.91	3.04	7.06	49.59	388.9
2012	1.73	2.69	7.31	51.02	397.1
2013	1.90	2.61	7.87	50.00	393.8
2014	2.12	2.12	7.94	43.90	374.0

Sources: the environmental and ecological monitoring bulletins of the Three Gorges Reservoir Area between 2009 and 2015.

**Table 2:** Definition of critical values and their error checks.

Item	I	II	III	IV
$\bar{\Delta }$	0.01	0.05	0.1	0.2
η	0.9	0.8	0.7	0.6

Data source: Liu, S.F.; Yi, L. Grey Information, 1st ed.; Springer-Verlag London Ltd.: the United States of America, 2006; pp. 279–280, ISBN 978-185233-995-1.

**Table 3:** Simulation results of $W_{1}$.

Year	$W_{1}$	${\hat{W}}_{1}$	$\varepsilon_{1}$
2008	5.58	5.58	0
2009	5.10	4.87	0.2306
2010	4.00	4.06	−0.0620
2011	3.04	3.39	−0.3485
2012	2.69	2.83	−0.1366
2013	2.61	2.36	0.2521
2014	2.12	1.97	0.1531

**Table 4:** Simulation results of $W_{2}$.

Year	$W_{2}$	${\hat{W}}_{2}$	$\varepsilon_{2}$
2008	5.93	5.93	0
2009	6.23	6.14	0.0914
2010	6.15	6.49	−0.3416
2011	7.06	6.86	0.1951
2012	7.31	7.26	0.0503
2013	7.87	7.68	0.1928
2014	7.94	8.12	−0.1787

**Table 5:** Simulation results of $W_{3}$.

Year	$W_{3}$	${\hat{W}}_{3}$	$\varepsilon_{3}$
2008	41.20	41.20	0
2009	41.30	45.98	−4.6850
2010	48.13	46.51	1.6178
2011	49.59	47.05	2.5445
2012	51.02	47.58	3.4351
2013	50.00	48.13	1.8695
2014	43.9	48.68	−4.7824

**Table 6:** Simulation results of $W_{4}$.

Year	$W_{4}$	${\hat{W}}_{4}$	$\varepsilon_{4}$
2008	404.6	404.6	0
2009	399.7	402.36	−2.6580
2010	400.5	398.29	2.2074
2011	388.9	394.27	−5.3683
2012	397.1	390.28	6.81534
2013	393.8	386.34	7.4587
2014	374	382.44	−8.4377

**Table 7:** Average relative errors and Grey correlation degrees of *W*_1_, *W*_2_, *W*_3_ and *W*_4_.

Item	$W_{1}$	$W_{2}$	$W_{3}$	$W_{4}$
$\bar{\Delta }$	0.0658	0.0253	0.0687	0.0141
η	0.9998	0.9923	0.9666	0.9664

**Table 8:** Prediction results of main water pollutant annual emissions in the Three Gorges reservoir area from 2015 to 2020.

Year	${\hat{W}}_{1}$ (10^8^ Tons)	${\hat{W}}_{2}$ (10^8^ Tons)	${\hat{W}}_{3}$ (10^4^ Tons)	${\hat{W}}_{4}$ (10^4^ Tons)
2015	1.64	8.59	49.24	378.57
2016	1.37	9.08	49.81	374.75
2017	1.14	9.60	50.38	370.96
2018	0.95	10.15	50.95	367.21
2019	0.79	10.74	51.54	363.50
2020	0.66	11.36	52.13	359.83
